# Gastrointestinal Basidiobolomycosis Accompanied by Liver Involvement: A Case Report

**DOI:** 10.5812/ircmj.14109

**Published:** 2014-08-17

**Authors:** Fardad Ejtehadi, Amir Anushiravani, Alimohammad Bananzadeh, Bita Geramizadeh

**Affiliations:** 1 Gastroenterohepatology Research Center, School of Medicine, Shiraz University of Medical Sciences, Shiraz, IR Iran; 2 Internal medicine Department, School of Medicine, Shiraz University of Medical Sciences, Shiraz, IR Iran; 3 Colorectal Research Center, School of Medicine, Shiraz University of Medical Sciences, Shiraz, IR Iran; 4 Transplant Research Center, School of Medicine, Shiraz University of Medical Sciences, Shiraz, IR Iran

**Keywords:** Fungal Infections, Basidiobolomycosis, Granuloma

## Abstract

**Introduction:**

Basidiobolomycosis is a rare disease that, unlike other fungal infections, affects immunocompetent individuals. It is caused by an environmental saprophyte named the fungus *Basidiobolus ranarum*. Basidiobolomycosis usually appears as a subcutaneous infection. GI basidiobolomycosis is an emerging disease, and the colon is the most frequent involved part of the GI tract.

**Case Presentation:**

The present study presents a middle-aged lady suffered from basidiobolomycosis with concomitant lesions in the cecum and liver involvement. This disease is extremely rare in adults and only a few cases have been reported so far.

**Conclusions:**

GI basidiobolomycosis is a very rare disease which resembles as an infiltrative, infectious, or inflammatory process. Concomitant liver and bowel involvement is extremely rare too. It is an aggressive disease which has a high mortality rate despite treatments like surgical resection and prolonged antifungal therapy.

## 1. Introduction

Basidiobolomycosis is a rare disease that, unlike other fungal infections, affects immunocompetent individuals. It is caused by an environmental saprophyte named the fungus Basidiobolus ranarum. Basidiobolomycosis usually appears as a subcutaneous infection. GI basidiobolomycosis is an emerging disease, and the colon is the most frequent involved part of the GI tract.

## 2. Case Presentation

In January 2012, a 41-year-old lady from Shiraz, a city in the south of Iran, Referred to Nemazee Hospital and sought medical attention. She was complaining of abdominal pain, nausea, and experienced significant weight loss since one month prior to the admission. Past medical history and personal history were not significant, and she had no specific risk factor exposure. On physical examination temperature was 38.5°C (101.3°F) and the signs of anemia, including pale conjunctiva were obvious. Also, she had mild generalized abdominal tenderness with no peritoneal sign. The patient was so cachexic and ill that hospital admission was advised; however, the main concern was the possible underlying malignancy versus infectious diseases like tuberculosis.

 Laboratory studies revealed high ESR titer and CRP level (80 mm/hour and 112 mg/L respectively), elevated alkaline phosphatase level and PMN, dominant leukocytosis accompanied by significant eosinophilia (12%). Cultures from different sites, stool examination and urinalysis were inconclusive, and tumor marker levels, including CEA, CA125, CA19-9, and AFP were within the normal ranges. Other paraclinical workup such as malaria smear, mammography, HBsAg, HCV Antibody, HIV and sputum Acid-fast bacilli smear were negative (Table 1).

**Table 1.** The Laboratory and Paraclinical Results of the Patient^a^

Parameter	P Value
**BS, mg/dL**	75
**BUN, mg/dL**	4
**Cr, mg/dL**	0.6
**Na, mEq/L**	133
**K, mEq/L**	4.8
**WBC, cells/mL**	14300
**Hb, g/dl**	8.7
**MCV, FL**	74
**Plt, /MCL**	138,000
**Neut, %**	67
**Lymph, %**	18
**Eos, %**	12
**Mono, %**	3
**Total protein, g/dL**	6.5
**Albumin, g/dL**	2.6
**ALT, IU/L**	8
**AST, IU/L**	9
**ALP, IU/L**	695
**T. Bilirubin, mg/dL**	0.5
**D. Bilirubin, mg/dL**	0.2
**Calcium, mg/dL**	8.8
**T3, pg/mL**	1.7
**T4, ug/dL**	11
**TSH, µIU/mL**	2.55
**LDH, IU/L**	310
**ESR, mm/h**	80
**CEA, ng/mL**	0.8
**CA125, U/mL**	13
**CA19-9, U/mL**	9.7
**αFP, ng/mL**	1.3
**HBS Ag**	Negative
**HCV Ab**	Negative
**HIV Ab**	Negative
**Blood culture**	No growth
**U/A**	Normal
**U/C**	No growth
**Stool exam**	Normal
**Sputum for AFB ×** **3**	Negative
**Wright, 2-ME**	Negative
**CRP, mg/dL**	113
**Mammography**	no malignant calcification

^a^ Abbreviations: BUN, Blood Urea Nitrogen; Cr, Creatinine; Na, Natrium (Sodium); K, Potassium; WBC, White Blood Cell; Hb, hemoglobin; MCV, Mean Corpuscular Volume; ALT, Alanine transaminase; AST, Aspartate transaminase; ALP, Alkaline Phosphatase; T3, Triiodothyronine; T4, Thyroxine; TSH, Thyroid Stimulating Hormone; LDH, lactate dehydrogenase; ESR, erythrocyte sedimentation Rate; CEA, Carcinoembryonic antigen; CA125, carcinoma antigen 125; CA 19-9, cancer antigen 19-9; αFP, Alpha-fetoprotein; HBS Ag, Hepatitis B surface Antigen; HCV Ab, Hepatitis C Virus Antibody; HIV, Human immune deficiency viruses; CRP, C-reactive protein.

**Figure 1.**
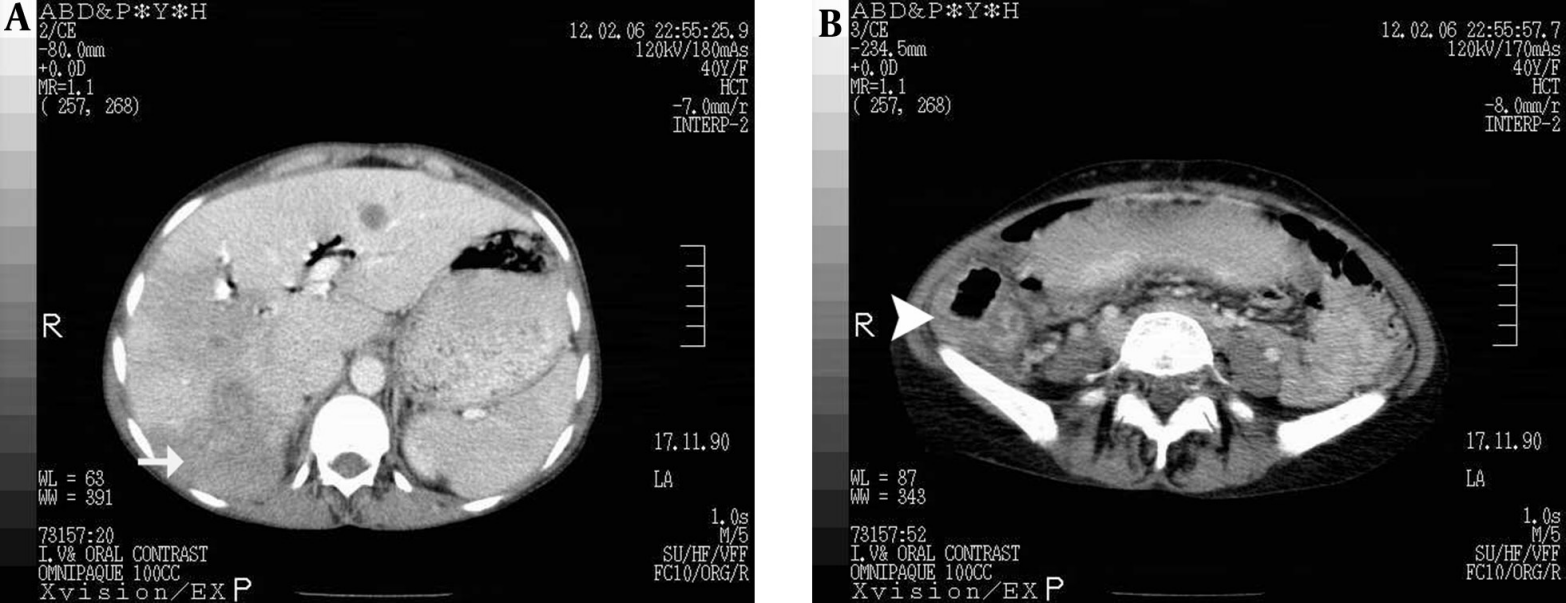
CT Scan of Abdomen A: CT Scan of the abdomen revealed hypoattenuating lesions in the liver (Arrow) and thickening of the cecal wall (Arrow head) in Figure 1 -B.

CT-scan of the abdomen and pelvic showed increased thickness of the cecum with infiltrative or tumoral process and round hypoattenuating lesions in the liver, supporting liver abscesses or metastasis with central necrosis (Figure 1 -A and Figure 1 -B).

**Figure 2.**
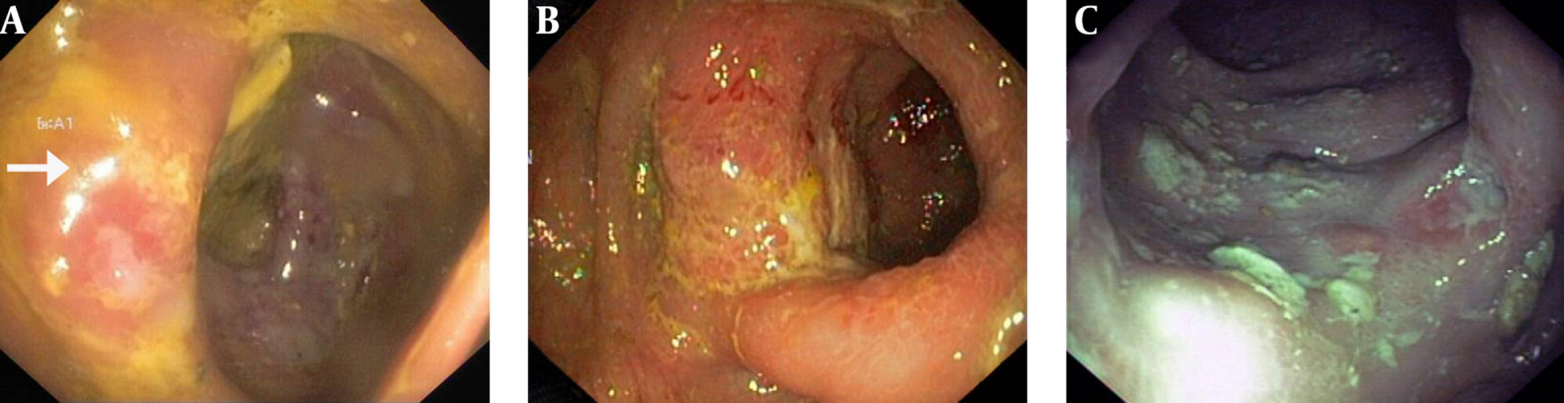
Colonoscopy Images of the Patient A: Ulcers in the cecum, covered with exudative tissues with surrounding edema that resembles an infiltrative process (Arrow). The same findings were reported in the second (Figure 2 -B) and third colonoscopy (Figure 2 -C).

The first colonoscopy study showed an infiltrative mass with nodularity and multiple ulcers involving the base of cecum and ileocecal valve (Figure 2 -A), but the pathology study revealed ulceration with acute and chronic inflammation and granulation tissue formation with negative tuberculosis PCR study. The same results were reported for the two other consecutive colonoscopies with similar morphologic findings (Figure 2 -B and Figure 2 -C).

**Figure 3.**
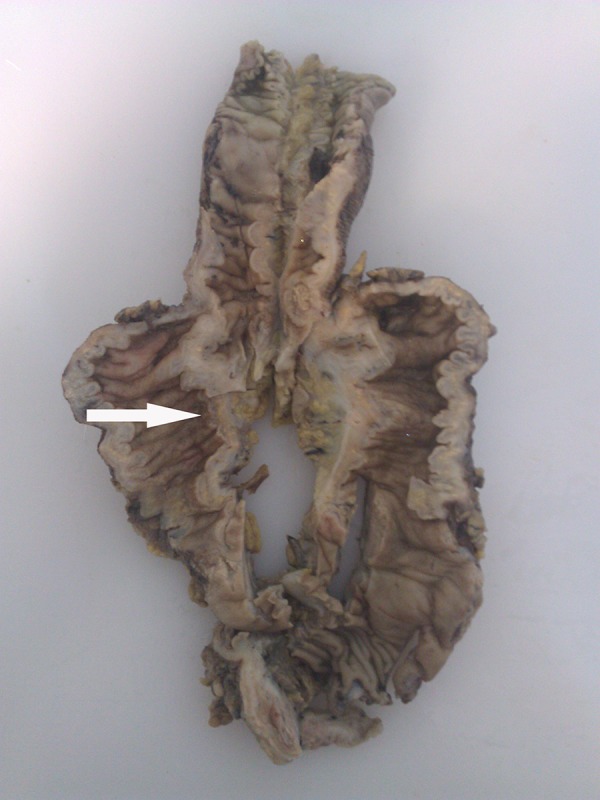
Gross Pathology of the Terminal Ileum and Right Colon Shows Ulceration in the Cecum (Arrow)

**Figure 4.**
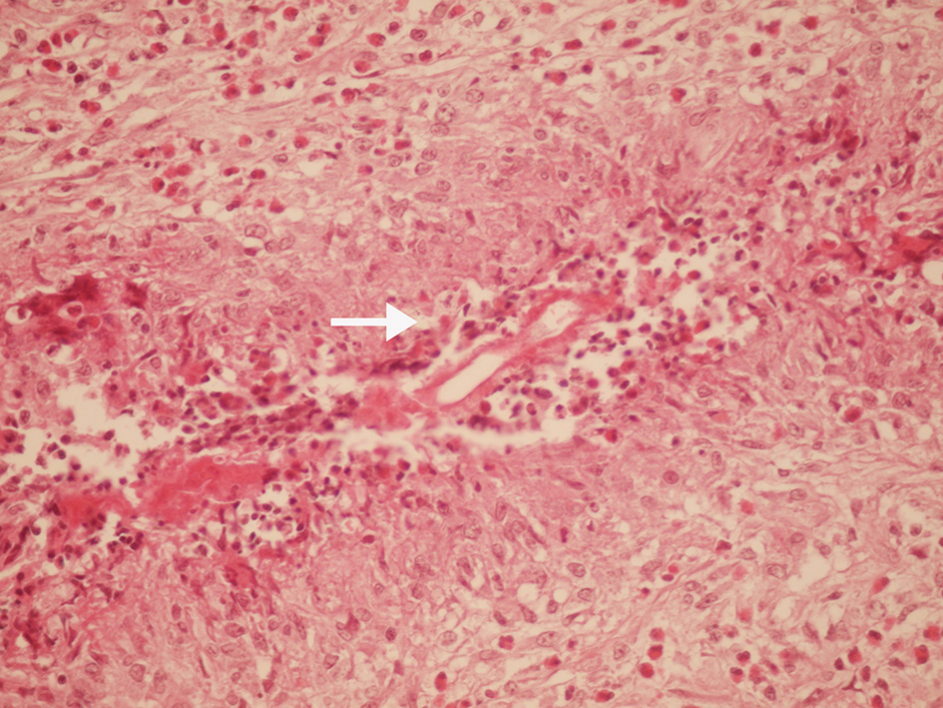
Necrotizing Granuloma, Eosinophilic Infiltration and Splendore-Hoeppli Phenomenon (Arrow)

Tru-cut liver biopsy from the hepatic lesions was done, which revealed granuloma with eosinophilic infiltration. Right hemicolectomy was done for the patient and pathology studies on the tissues of the resected cecum, and terminal ileum (Figure 3) revealed the presence of the fungal elements, necrotizing granuloma, eosinophilic infiltration and Splendore-Hoeppli phenomenon (the deposition of amorphous, eosinophilic, hyaline material around pathogenic organisms). Therefore (Figure 4), the diagnosis of GI basidiobolomycosis was confirmed.

**Figure 5.**
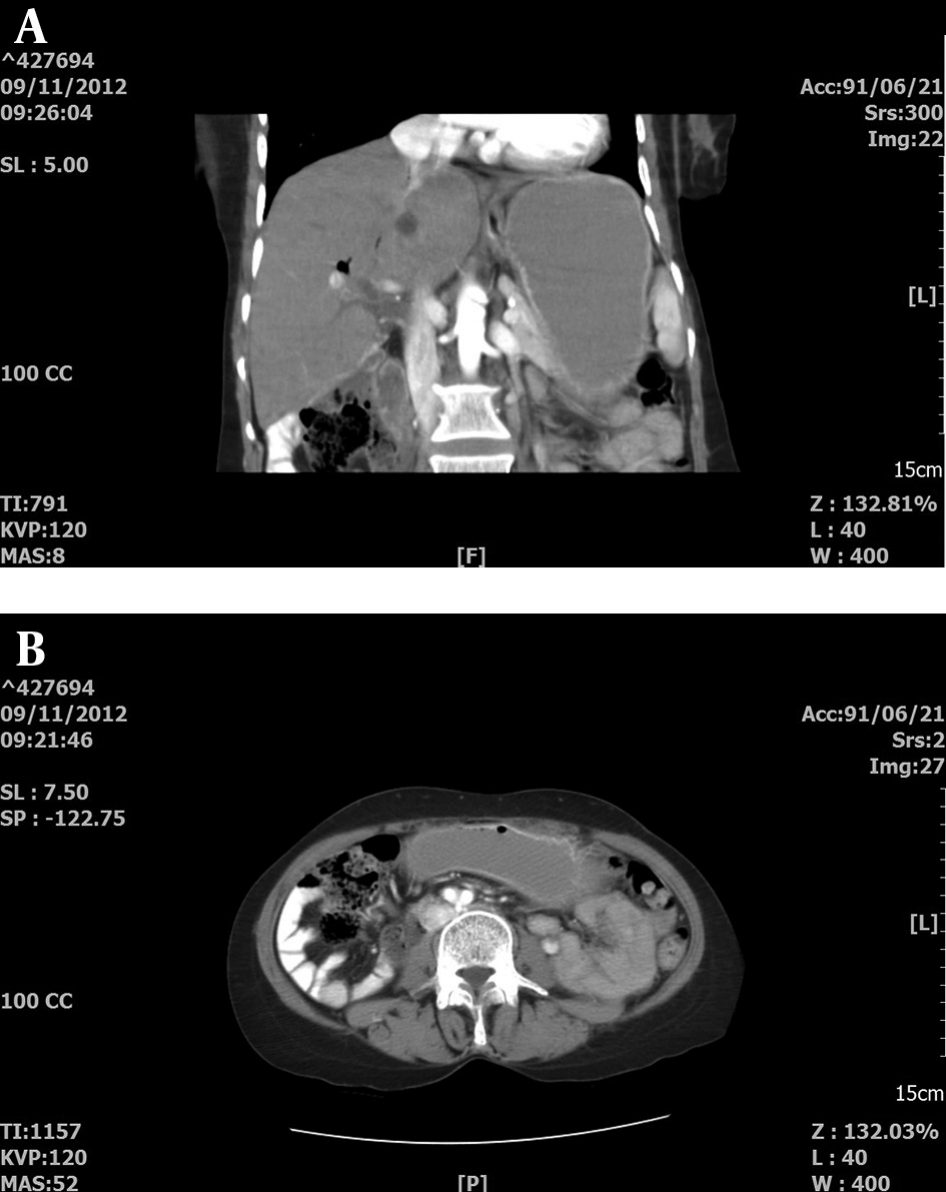
Post Treatment Ct Scan of the Abdomen CT Scan of the Abdomen of the Patient Taken 2 Months After End of Treatment With no Evidence of Active Disease in the Liver (A) and in the Anastomosis Area (B)

Finally, the patient was treated with itraconazole 200 mg twice a day for 4 months. ESR titer and alkaline phosphatase level returned to normal and eosinophilia resolved within the first 3 weeks of treatment. The patient health status showed significant improvement, and no evidence of active liver lesions was detected in follow-up imaging study (Figure 5 -A and Figure 5 -B). In the next year of follow-up, the patient was healthy and symptom free.

## 3. Discussion

Zygomycosis is considered as a large group of infections that are caused by the fungi of the zygomycota class and Entomophthorales order (1). Basidiobolus ranarum, filamentous fungi, is one the three species of the Entomophthorales order and belongs to family Basidiobolaceae and genus Basidiobolus (2). Basidiobolus ranarum was first isolated from the decaying plants in the United States in 1955 (3). The fungus is present in the intestine of insectivorous bats, fish, reptiles, and amphibians (4). The organism is found worldwide (3), but the largest number of cases of basidiobolus infection has been reported from tropical and subtropical regions (1-3).

Basidiobolomycosis is a very rare disease that, unlike other fungal infections affects immunocompetent patients (2, 4-7), and no underlying disease seems to predispose to basidiobolomycosis (1).

 Basidiobolus ranarum usually causes cutaneous or subcutaneous infections (8). The first case report of cutaneous zygomycosis due to Basidiobolus ranarum was described in 1956 (1) and the first culture proven disease was reported in 1978 (9). Only 160 cases of cutaneous infections have been reported so far (9). As the fungus is found globally in the environment, the surprisingly low prevalence of the disease could be due to immunity from previous subclinical infection or lack of a pathogenic strain (1).

The route of transmission is still unclear (1), but presumed to be through the insect bite or fungus exposure via minor trauma to the skin (4). The disease begins with a small papule on the site of a scratch or skin puncture (1). The most common initial presentation is an indolent infection of the subcutaneous tissues with a firm, non-ulcerated lesion (2). Gastrointestinal involvement in basidiobolomycosis is extremely rare. Few cases of gastrointestinal infection had been diagnosed until 2001 (7) and only about 44 cases have been reported in immunocompetent adults and children worldwide, until now (6-8, 10). GI basidiobolomycosis may present with fever and abdominal symptoms accompanied by eosinophilia and high ESR titer (3, 5, 7), and a mass-like lesion in the colon that resembles colon cancer (3, 5, 10), infections like tuberculosis, inflammatory processes such as inflammatory bowel disease (Crohn’s disease or infective colitis) (7) or other infiltrative lesions of the colon (10).

 Nevertheless, concomitant liver involvement is an extremely rare condition, and only a few cases (4 cases) have been reported in adults. The diagnosis of GI basidiobolomycosis is very difficult and requires a high index of suspicion. The gold standard of diagnosis is culture (7, 8, 11), with a yellow-grayish colonies (12) and characteristic waxy growth (2). However, most cases that have been reported in the literature have been diagnosed on the basis of the characteristic histopathological features (5, 9, 11), which are eosinophilic infiltration, Splendore-Hoeppli phenomenon and the fungal morphology (3, 7, 8, 12). The area of Splendore-Hoeppli phenomenon is composed of thick, hyalinized, eosinophilic material that surrounds the fungal hyphae (7). Non-caseating granulomas are often present (4, 7, 12, 13), albeit not characteristic.

 GI basidiobolomycosis is an aggressive disease. Surgical resection and prolonged administration of antifungal agents are the corner stones of treatment (2-4, 7, 10-14). Unfortunately, the basidiobolomycosis has a 20% mortality rate despite all of the therapeutic measures (10). The choice and duration of antifungal therapy are not well-established. Itraconazole, fluconazole, ketoconazole, miconazole, (4) and variconazole (12) all have in vitro activity against Basidiobolus ranarum(4). Nevertheless, itraconazole is the most frequently administrated antifungal treatment (2-4, 7, 11-13), with the preferred dose of 100 mg twice daily (2). The duration of treatment varies from 4 months up to 1-2 years with an average of 8 months (2, 4, 11, 12). There are some reports of successful treatment with Posaconazole (6, 12) but treatment response to amphotericin B has been reported as unsatisfactory in some cases (3, 7).

 Basidiobolomycosis is a very rare fungal disease in immunocompetent patients with no specified predisposing factor, which usually causes cutaneous infections. Gastrointestinal involvement by this fungus is very rare and the disease resembles as an infiltrative, infectious or inflammatory process of small bowel and colon. Concomitant liver and bowel involvement is an extremely rare condition, and only a few cases have been reported in adults. The characteristic histopathological features are eosinophilic infiltration, Splendore-Hoeppli phenomenon and the fungal morphology. GI basidiobolomycosis is an aggressive disease with 20% mortality rate, which its treatment requires surgical resection and prolonged antifungal therapy. Therefore, physicians should be vigilant about the possibility of gastrointestinal basidiobolomycosis in the presence of any infiltrative lesions or eosinophilic granuloma and abscess in the absence of specific etiology in immunocompetent patients.
